# Not All Pulmonary Densifications Are COVID-19: A Case Report About Lemierre's Syndrome

**DOI:** 10.7759/cureus.15984

**Published:** 2021-06-28

**Authors:** Fátima Costa, Margarida Matos Bela, Inês Ferreira, Catarina Cidade Rodrigues, Adriana América Silva

**Affiliations:** 1 Internal Medicine, Centro Hospitalar Tâmega e Sousa, Penafiel, PRT; 2 Endocrinology, Centro Hospitalar Tâmega e Sousa, Penafiel, PRT; 3 Intensive Care Unit, Centro Hospitalar Tâmega e Sousa, Penafiel, PRT

**Keywords:** lemierre’s syndrome, jugular vein thrombosis, fusobacterium, anaerobic bacteraemia, sars cov2

## Abstract

Lemierre's syndrome is a rare and a life-threatening disease characterized by anaerobic bacteraemia associated with thrombosis of the internal jugular vein. Odynophagia, otalgia, odontalgia, dyspnoea, cough and fever are the most frequent manifestations. We describe a case of a 37-year-old woman who was admitted to the emergency room due to fever, odynophagia, dyspnoea, myalgia, and pleuritic chest pain. She had hypoxaemia and increased systemic inflammatory markers. The chest CT showed parenchymal densification compatible with severe acute respiratory syndrome coronavirus infection, although all three polymerase chain reaction testing were negative. The neck CT showed occlusion of the left cervical internal jugular vein. She was treated with antibiotics and was discharged. With the reported clinical case the authors intend to clarify the importance of differential diagnosis and the diagnosis of other infectious respiratory conditions at the time of a global pandemic.

## Introduction

Lemierre’s syndrome is a rare and a life-threatening condition described for the first time in 1900 by Courmont. In 1936 [1], André Lemierre published a series of 20 cases. After the introduction of antibiotics and increased use of penicillin for the treatment of oropharyngeal infections, the incidence of Lemierre’s syndrome has been decreasing, currently being a rare diagnosis, with an estimated incidence of one in 1 million in a Danish study [2]. Mortality during the pre-antibiotic era was 90% [1-3], and nowadays is 5% [4].

The pathogenesis of the disease is thought to be due to the invasion of the oropharynx mucosa by *Fusobacterium necrophorum*. It is usually diagnosed as a superinfection, secondary to pharyngitis, otitis, mumps, sinusitis, or dental infection, or even associated with Epstein Barr infection [5]. The proximity to the internal jugular vein facilitates the dissemination of fusobacteria from the peritonsillar and paratracheal space into the vessels with consequent thrombus formation in this vessel, leading to bacteremia and septic embolism with distant organ involvement. Pleuropulmonary involvement is the most prevalent presentation, occurring in 95% of cases [5,6].

It usually affects previously healthy adolescents and young adults. The diagnosis is based on the isolation of the infectious agent in blood cultures as well as CT with contrast. Pulmonary emboli are also common [2]. Treatment is based on antibiotic coverage of beta lactamase-producing agents and it must be maintained until clinical and/or imagiological resolution. Usually there is the need for IV treatment for at least two weeks [2]. The hypocoagulant treatment decision remains controversial [2].

## Case presentation

**Figure 1:**
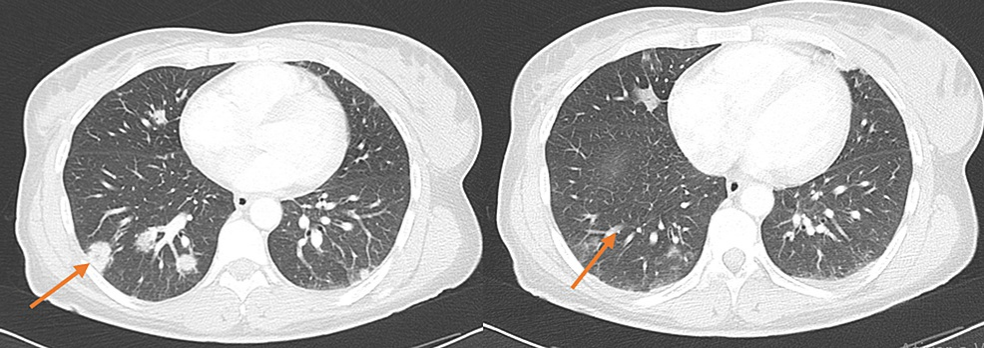
CT on admission showing parenchymal densification (arrows)

A 37-year-old woman was admitted to the ER due to fever with five days of evolution (maximum temperature of 42.5ºC) along with odynophagia. She had been previously treated with paracetamol 1 g tid and clarithromycin without complete symptomatic relief. Due to clinical worsening with new-onset exertional dyspnoea, myalgia, and pleuritic chest pain, she returned to the ER. She had hypoxaemia (pO_2_ 76 mmHg), increased systemic inflammatory markers (leukocytosis 13,680/µL, neutrophilia 11,425/µL, C-reactive protein 297.1 mg/L), and thrombocytopenia of 72,000/µL. Chest CT (Figure 1) revealed areas of peripheral parenchymal densification in different lobes of the lungs, predominantly in the pulmonary bases, compatible with infection by COVID 19 with a small amount of bilateral pleural fluid accumulation. Past medical history included an episode of foetal death at 25 weeks of gestation due to placental pathology and iron-deficiency anaemia with a baseline haemoglobin value of 11.5 g/dL. She was taking ferrous sulphate 329.7 mg id. She denied contacts with any animals, consumption of unpasteurized milk, recent travels, consumption of herbal medicines, teas or illicit drug use.

Given the CT findings as well as the current pandemic context, a high probability of infection by SARS-CoV2 (severe acute respiratory syndrome coronavirus 2) was considered. She started empirical therapy with ceftriaxone, azithromycin and hydroxychloroquine (recommended at the time in patients with SARS-CoV2 infection). In total, three SARS-CoV2 searches were performed on different days, all negative. On the sixth day of hospitalization, a chest CT scan was repeated and it revealed some rounded densifications, with no images of cavitation. A neck CT was performed and it showed thickening of the soft tissues in the left peri-tonsillar region, bulging of the posterolateral wall of the oropharynx, left jugulodigastric adenopathy, occlusion of the left cervical internal jugular vein that presents with emphysematous component adjacent to the anterior wall, and partial obliteration of the left para-pharyngeal space.

These findings could be compatible with pneumonia, and given the negative results in the SARS CoV2 tests, the most common respiratory agents were tested (Table 1).

**Table 1:** Laboratory tests performed during admission

Agent	Result	Interpretation/reference value
Respiratory syncytial virus	Negative	-----------------------
Adenovirus	Negative	-----------------------
Influenza A and B	Negatives	-----------------------
Parainfluenza 1-3	Negatives	-----------------------
*Legionella pneumophila* urinary antigen	Negative	-----------------------
*Streptococcus pneumoniae* urinary antigen	Negative	-----------------------
Hepatitis B surface antigen	Non-reactive	-----------------------
Hepatitis B surface antibody	959.91 mUI/mL	Protection ≥10 mUI/mL
Hepatitis B core antibody	Non-reactive	-----------------------
Hepatitis C antibody	Non-reactive	-----------------------
Antibodies against HIV-1 and HIV-2	Non-reactive	-----------------------
*Coxiella burnetii* (Q-Fever) antibody IgG, phase I and II	<200 titers	Positive ≥200
*Coxiella burnetii* (Q-Fever) antibody IgM, phase I and II	<50 titers	Positive ≥50
Brucella IgG antibody	Negative	------------------------
Brucella IgM antibody	Negative	------------------------
Epstein Barr IgM viral capsid antibody	0.02 index	Negative <0.12
Epstein Barr IgG viral capsid antibody	1.21 index	Positive >0.20
Epstein Barr IgG nuclear antibody	2.94 index	Positive >0.20

 

A thoracocentesis was performed and the drained pleural fluid was sterile, negative for acid-resistant bacilli and mycobacteriology. No immunophenotypical changes were detected and cytology was negative for malignant cells. There was a predominance of polymorphonuclear cells, normal glucose, and negative adenosine deaminase and the Light’s criteria were met, so it was considered an exudate pleural effusion.

An autoimmune study was performed (Table 2).

**Table 2:** Autoimmune study performed during admission

Parameter	Result	Interpretation/reference value
Erythrocyte sedimentation rate	105 mm	0-20 mm
Rheumatoid factor	<20 UI/mL	<20 UI/mL
Complement C3	145 mg/dL	90-180 mg/dL
Complement C4	28 mg/dL	10-40 mg/dL
Immunoglobulin A	303 mg/dL	70-400 mg/dL
Immunoglobulin G	1501 mg/dL	700-1500 mg/dL
Immunoglobulin M	125 mg/dL	40-230 mg/dL
Immunoglobulin D	1.5 mg/dL	<4 mg/dL
Anti-nuclear antibodies (Ena’s, FEIA)	0.40 Ratio	Negative <0.7
Anti-nuclear antibodies (anti-ds DNA)	2.10 UI/mL	Negative <10 UI/mL
Beta-2 glycoprotein 1 antibody IgM	2.10 UI/mL	Negative <7 UI/mL
Beta-2 glycoprotein 1 antibody IgG	1.20 UI/mL	Negative <7 UI/mL
Beta-2 glycoprotein 1 antibody IgA	<4.0 UQ	<20 UQ
Anti-cardiolipin antibody IgM	132.0 MPL-U/mL	Positive >40 MPL-U/mL
Anti-cardiolipin antibody IgG	13 GPL-U/mL	Weak positive 10-40 GPL-U/mL
Anti-cardiolipin antibody IgA	10.2 UQ	<20 UQ
Anti-neutrophil cytoplasmic antibody (PR3)	0.7 UI/mL	Negative <2 UI/mL
Anti-neutrophil cytoplasmic antibody (MPO)	0.2 UI/mL	Negative <3.5 UI/mL
Angiotensin-converting enzyme (ACE)	25 U/L at 37degrees	8-76 U/L
Lupus anticoagulant	Negative	-----------------------------------

Hypocoagulant treatment initially consisted of low-molecular-weight heparin and subsequently rivaroxaban 15 mg once daily, for the total duration of four months.

After 14 days of treatment, cervical-thoracic CT was repeated, which demonstrated persistence of the occlusion in the left internal jugular vein, persistence of the pleural effusion, with thickening of the pleural leaflets, probably evidencing an empyema. There were nodular opacities at the level of the middle and right lower lobes. Two of them showed cavitation aspects, probably corresponding to sequelae of septic emboli. Another opacity was observed in the upper left lobe, with an almost entirely gaseous component, traducing probable cavitation.

The patient completed a 20-day course of antibiotic therapy, with a favourable outcome. She was discharged and a follow-up internal medicine appointment was scheduled.

**Figure 2:**
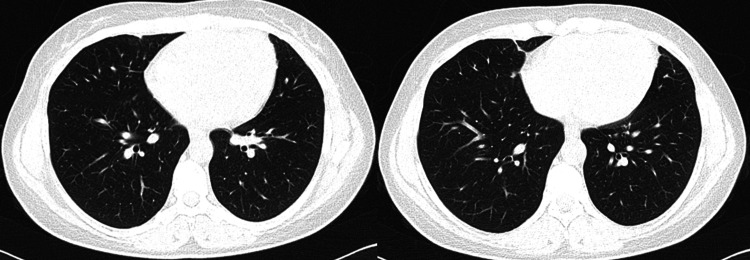
CT after six months without parenchymal densification

After six months, she repeated chest CT, which showed no cavitated lesions and no pleural effusion. There was a marked improvement of the previously observed basal atelectasis. Anticardiolipin antibodies were negative (Figure 2).

## Discussion

This clinical case is that of a young woman with no significant comorbidities who was affected by Lemierre's syndrome. The onset of her condition suggested oropharyngeal infection (high fever, preceded by odynophagia in about five to 12 days [2]), but with an atypical evolution, despite antibiotic treatment.

Given the symptoms and signs of worsening lower respiratory infection presented by this patient (fever, dyspnoea, hypoxaemia and pleuritic pain), as well as the findings on chest CT, the hypothesis of SARS-CoV2 pneumonia was considered taking into account the epidemiological context. After exclusion of COVID 19 infection by repeated polymerase chain reaction testing, the search for another microbiological agent was expanded, including other viruses. Given the past medical history, an autoimmune diseases study was performed and only the anti-cardiolipin antibody IgM positive and IgG weak positive were detected, which were negative after 12 weeks.

The revision of the symptomatic chronology and a neck CT scan showing very suggestive findings led to the conclusion of the diagnosis of acute tonsillitis complicated by septic jugular vein thrombosis (Lemierre’s syndrome), with consequent pulmonary embolization, formation of pulmonary abscesses and sepsis.

Initially, no agent was isolated in the blood cultures performed because the first blood cultures did not include culture for anaerobic agents, and the latter ones were performed when the patient was already under antibiotic therapy.

We decided to start broad-spectrum empirical antibiotics with anaerobic coverage using piperacillin-tazobactam, as well as prompt hypocoagulation.

The onset of hypocoagulation is still controversial. Some authors recommend it due to the faster resolution of thrombosis and bacteremia, thus preventing the development of new metastatic foci [2]. In this case, it was considered taking into account the high thrombotic risk presented by the patient [7]. Other authors consider that hypocoagulation should only be performed when thrombosis is extended to the cerebral vessels or if there is no symptomatic improvement with antibiotic treatment, since hypocoagulant therapy facilitates the penetration of antibiotics in the septic embolus [2].

## Conclusions

Delayed recognition of this syndrome increases the risk of disease severity and mortality. This case represents how the findings on chest CT, initially overlapping the findings of SARS-CoV2 pneumonia, delayed the investigation for other agents and pathologies with consequent clinical worsening until the beginning of appropriate antibiotic strategy.

The description of this case intends to alert to the difficulty in diagnosing other infectious respiratory conditions at the time of a global pandemic, and to enhance the role of actively searching for alternative diagnoses and overall assessment of the patient.
